# High conductance potassium channels activation by acid exposure in rat aorta is endothelium-dependent

**DOI:** 10.1186/s13104-015-1422-3

**Published:** 2015-09-19

**Authors:** Andrea Carla Celotto, Verena Kise Capellini, Agnes Afrodite Sumarelli Albuquerque, Luciana Garros Ferreira, Ana Paula Cassiano Silveira, Tales Rubens de Nadai, Paulo Roberto Barbosa Evora

**Affiliations:** Department of Surgery and Anatomy, Laboratory of Endothelial Function, School of Medicine, University of São Paulo, Av. Café, 3000, Ribeirão Preto, SP Brazil; Av. Bandeirantes, 3900, HC-FMRP, 9o. andar, Ribeirão Preto, SP 14.049-900 Brazil

**Keywords:** Potassium channels, Endothelium, Paxilline, Rat aortic rings

## Abstract

**Background:**

We investigated, previously, the mechanism by which extracellular acidification promotes relaxation in rat thoracic aorta. These studies suggested that extracellular acidosis promotes vasodilation mediated by NO, K_ATP_ and SK_Ca_, and maybe other K^+^ channels in isolated rat thoracic aorta. This study was carried out to investigate the paxilline-mediated hyperpolarization induced by acid exposure.

**Results:**

The relaxation response to HCl-induced extracellular acidification (7.4–6.5) was measured in rat aortic rings pre-contracted with phenylephrine (PE, 10^−6^ M). The vascular reactivity experiments were performed in endothelium-intact and denuded rings, in the presence of paxilline (10^−6^ M), which is an inhibitor of high calcium conductance potassium BK_Ca_ channels. In rings with endothelium, paxilline inhibits relaxation, triggered by acidification at all pH values lower than 7.2 and had no effect on rings without endothelium, showing that the activation of BK_Ca_ is endothelium-dependent.

**Conclusion:**

High conductance potassium channel activation induced by acid exposure is endothelium-dependent.

## Background

We investigated, previously, the mechanism by which extracellular acidification promotes relaxation in rat thoracic aorta. The extracellular acidosis failed in inducing any changes in the vascular tone of rings pre-contracted with potassium chloride (KCl). However, it caused endothelium-dependent, and endothelium-independent, relaxations in rings pre-contracted with PE. These relaxations were inhibited by L-NAME, apamin, and glibenclamide, but not by indomethacin. These results suggested that extracellular acidosis promotes vasodilation mediated by NO, K_ATP_ and SK_Ca_, and maybe other K^+^ channels in the isolated rat thoracic aorta [1]. In rings with endothelium, paxilline inhibits relaxation induced by acidification at all pH values lower than 7.2. Otherwise, paxilline had no effect on rings without endothelium, showing that the activation of BK_Ca_ is endothelium-dependent. After an extensive literature search, we assumed this data as original. Therefore, the rationale of the present study was to test a hypothesis that the relaxation induced by extracellular acidification includes the activation of high conductance BK_Ca_ potassium channels.

## Methods

The experimental procedures and animal handling were reviewed and approved by the Institutional Animal Care Review Board (CETEA—Ethics Committees of Animal Experiments of the Faculty of Medicine of Ribeirão Preto—University of São Paulo). This investigation conforms with the Guide for the Care and Use of Laboratory Animals published by the US National Institutes of Health (NIH Publication No. 85-23, revised 1996). Before the experiments, the rats were housed under standard laboratory conditions (12 h light/dark cycle at 21 °C), with free access to food and water. Male Wistar rats (230–280 g) were anesthetized with thiopental sodium (40 mg/kg, intraperitoneal injection), followed by a laparotomy for exsanguination via the abdominal aorta and a thoracotomy for thoracic aorta harvesting. The thoracic aorta was carefully dissected free of connective tissue and immediately immersed in Krebs. For the vascular reactivity studies, Krebs solution with the following composition (mM) was employed: NaCl—118.0, KCl—4.7, CaCl_2_—2.5, KH_2_PO_4_—1.2, MgSO_4_—1.66, glucose—11.1, NaHCO_3_—25.0 (pH 7.4).

The thoracic aorta was cut into rings (4–5 mm in length). The endothelium was removed from some rings by gently rubbing the intimal surface of the blood vessel with a pair of watchmaker’s forceps. This method removes the endothelium, but it does not affect the ability of the vascular smooth muscle to contract or relax. Then, these rings were placed in isolated organ baths (10 mL) filled with Krebs solution, maintained at 37 °C, and bubbled with 95 % O_2_/5 % CO_2_ (pH 7.4). Each arterial ring was suspended by two stainless steel clips placed through the lumen. One clip was anchored to the bottom of the organ bath, while the other was connected to a strain gauge for measurement of the isometric force with the aid of the Grass FT03 equipment (Grass Instrument Company, Quincy, MA, USA). Each ring was stretched to a resting tension of 1.5 g and allowed to equilibrate for 60 min. During this period, tissues were washed every 15 min. The efficacy of the procedure for endothelium removal was confirmed by the lack of relaxant effects induced by acetylcholine (10^−6^ M) in rings pre-contracted with PE (10^−6^ M—EC_80_). Endothelium was considered to be present when the Ach-induced relaxation was at least 80 %. Then, each ring was washed and re-equilibrated for 30 min. Aortic rings were then pre-contracted with PE (10^−6^ M), and pH-response curves were obtained after a stable plateau was reached. The pH-response curves allowed assessment of the relaxation response to HCl-induced (1N) extracellular acidification (7.4, 7.3, 7.2, 7.1, 7.0, 6.9, 6.8, 6.7, 6.6, 6.5). For this purpose, an electrode (Analion G2134, Ribeirão Preto, SP, Brazil) connected to a pH-meter (MS tecnopon MPA 210, Piracicaba, SP, Brazil) was immersed in the organ bath, enabling real-time analysis of the Krebs solution pH, each point of the curve was obtained by adding a sufficient volume (approximately 2–3 μL) of HCl to the preparation, in order to decrease the pH in 0.1 units. The time elapsed between consecutive additions of acid was 1–2 min, which was necessary for observation of the effect of pH-changes and achievement of the plateau. Considering that the pH curve ranged from 7.4 to 6.5, the total final volume of HCl added to the organ bath was about 30 μL. The paxilline added to the bath was 10^−6^ M. Preparations were kept in contact with the drugs for an incubation period of 30 min. Phenylephrine (PE) and paxilline were purchased from Sigma Chemical Company (St. Louis, MO, USA). All the drugs were prepared with distilled water.

The results are shown as mean ± SEM. In the relaxation study, the changes in vascular tension are expressed as a percent of relaxation about the maximal contraction achieved by EC_80_ PE-stimulated contraction, a convention that corrects interanimal variability. Statistical analysis was performed using two-way repeated-measures ANOVA and Bonferroni post-test (Prism 4.0, GraphPad Software Inc, San Diego, CA, USA). Values were considered to be statistically significant at p values less than 0.05.

## Results

**Fig. 1:**
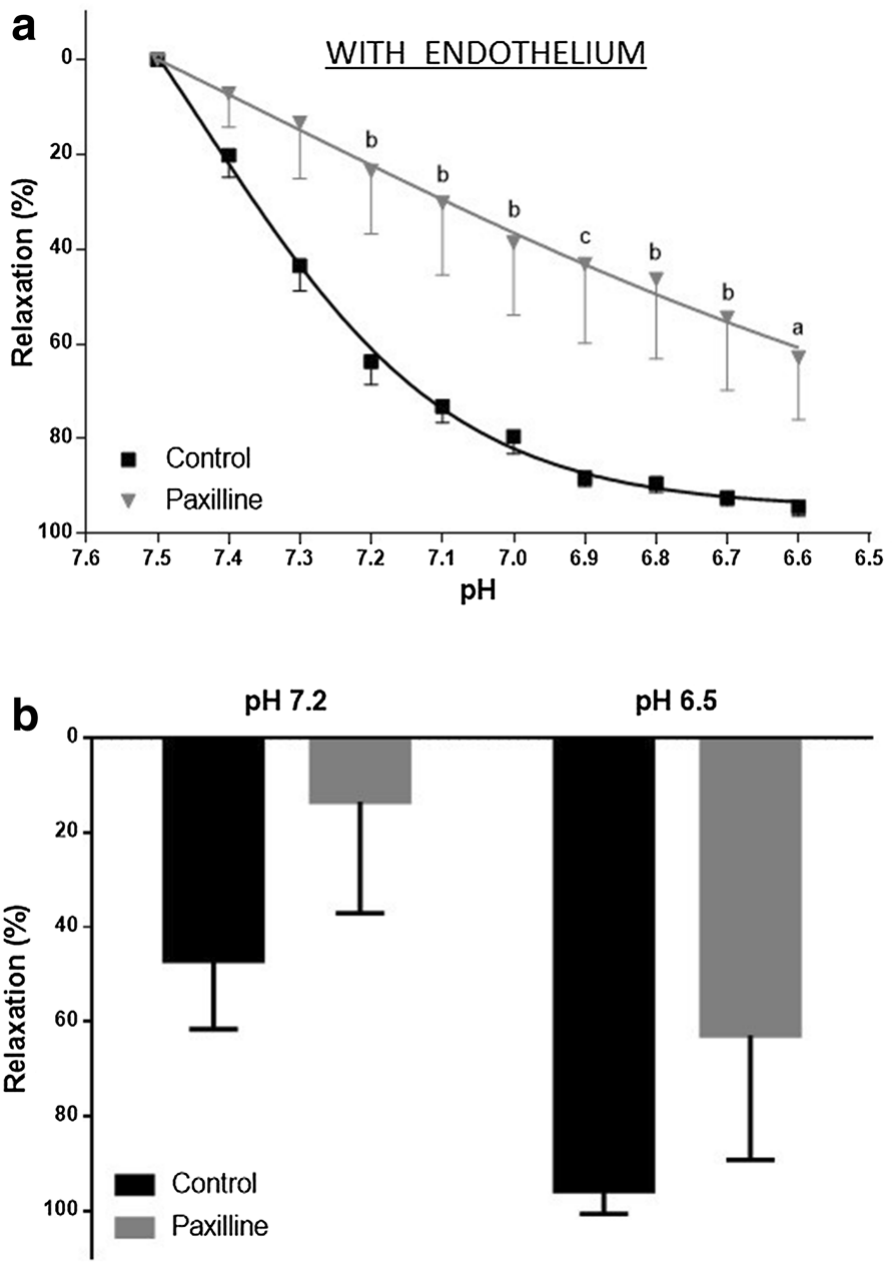
**a** pH-response curves for acidification in rat aortic rings with endothelium precontracted with PE (10^−6^ M) in the absence and presence of paxilline (10^−6^ M); **b** relaxation at pH 7.2 and pH 6.8. Data represent mean ± SEM (n = 6), ap <0.01, bp <0.001 e cp <0.05 (versus control paxilline), two-way ANOVA, Bonferroni post-test

In rings with endothelium precontracted with PE (10^−6^ M), paxilline inhibits relaxation, induced by HCl acidification at all pH values lower than 7.2 (n = 6; p < 0.05) (Fig. 1).

**Fig. 2:**
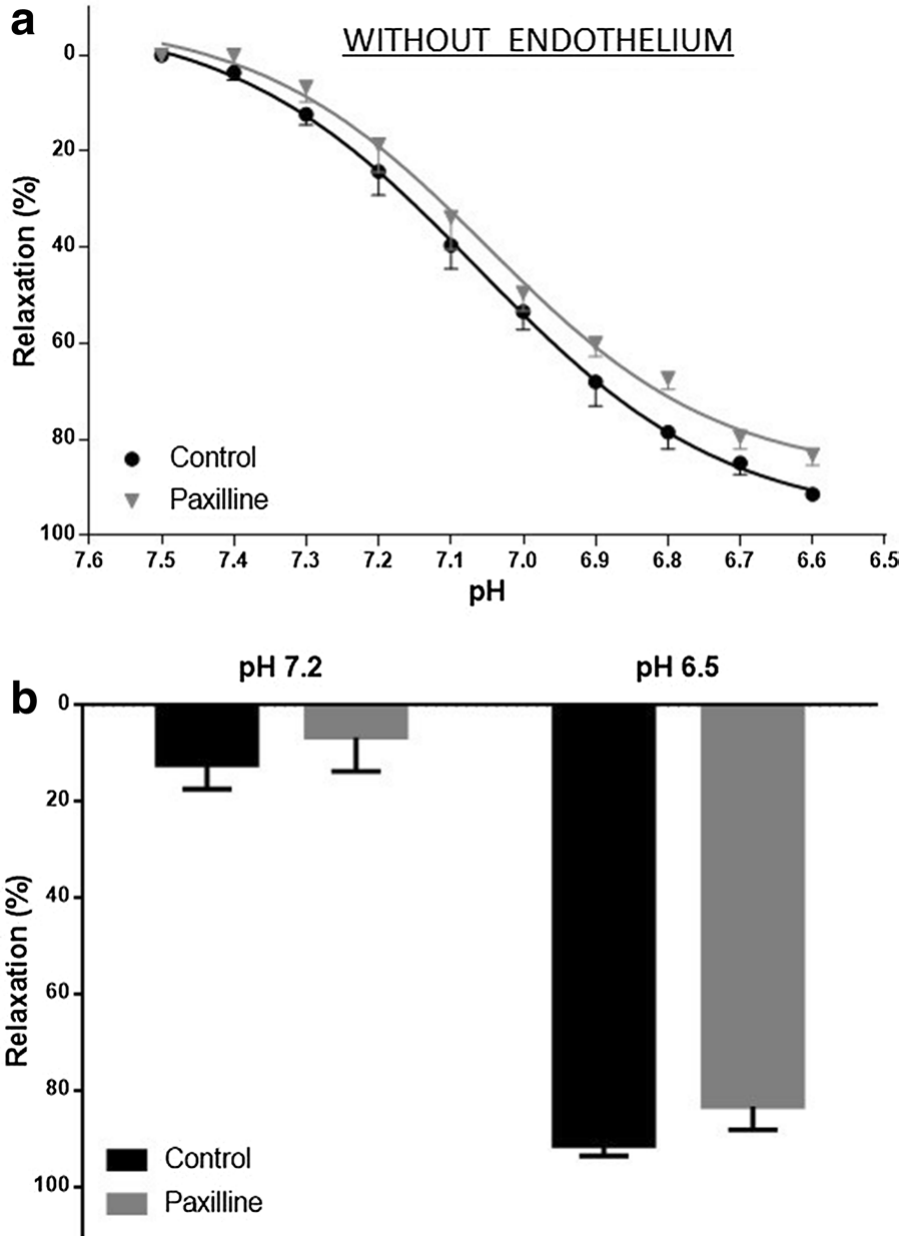
**a** pH-curves in response to acidification of rat aortic rings without endothelium, precontracted with PE (10^−6^ M) in the absence and presence of paxilline (10^−6^ M). **b** Relaxation at pH 7.2 and pH 6.8. Data represent mean ± SEM (n = 6), two-way ANOVA, Bonferroni post-test

In rings without endothelium precontracted with PE (10^−6^ M), paxilline did not inhibit the relaxation induced by HCl acidification at all pH values (n = 6; p < 0.05) (Fig. 2).

## Discussion

Several types of K^+^ channels have been identified in the vascular smooth muscle. These channels had been shown to be involved in the vasodilation seen in cerebral circulation under acidosis condition. K_ATP_ and K_Ca_ are the most important potassium channels engaged in the relaxant response [2–6] in the presence of decreased extracellular pH. It is known that high potassium concentrations can inhibit the activity of all potassium channels [4, 7].

To confirm the involvement of this two potassium channels in acidosis-induced vasodilation, we previously tested the specific channels K_ATP_ and K_Ca_ under acidosis condition concluding that their vascular beds actions were endothelium-independent. Vasodilation in response to acidosis was completely abolished when only one of these channels was blocked. The inhibitory effect of glibenclamide or apamin on vasodilation HCl-induced, was potenciated by endothelium removal. The association between L-NAME and specific potassium channels blockers, inhibited the HCl-relaxation almost a hundred percent. [1]. This data is suggestive that hyperpolarization and NO/cGMP has some “cross-talked” mechanism. It has been shown that the vasodilator response to NO in rat middle cerebral artery is mediated by activation of K_Ca_ channels via a cGMP- independent pathway [8], a finding that is in accordance with our results about the important role of NO and K_Ca_ channel activity in acidosis.

To evaluate the involvement of BK_Ca_, this study used paxilline [9], because although some studies have used charybdotoxin as a blocker of BK_Ca_ [2, 10], it is known that this drug is not unique to this channel [11–14]. The present studies, using the blocker paxilline, showed an interesting data: the effect of acidification mediated by potassium channel type BK_Ca_ is dependent on the endothelium. However, it is prudent to consider the fact that paxilline inhibit the relaxation only in rings with endothelium, suggesting that the opening of these channels should be dependent on some endothelium-derived factor. From this point of view, the paxilline BK_Ca_ channel blocker effect, by itself, should not participate in the relaxation induced by acidification. In addition, it is possible to speculate that the endothelium role is dependent on calcium conductance, since the acidosis-induced relaxation blocked by apamin (small conductance Ca^2**+**^-activated K^**+**^ channel blocker—SK_Ca_) was endothelium-independent, and paxilline (a high calcium conductance BK_Ca_) was endothelium-dependent.

The present study demonstrated the contribution of BK_Ca_ for dilation evoked by decreased pHo and paxilline proved to be useful as a pharmacological tool. For example, recent study about the vasorelaxant effect of cilostazol a selective inhibitor of type III phosphodiesterase (PDE3), was not affected by removal of the endothelium and the results were quite similar to the decreased pHo effect. Application of a nitric oxide synthase inhibitor and a small conductance Ca^2+^-activated K^+^ (SK_Ca_) channel inhibitor did not affect cilostazol-induced vasorelaxation, which was endothelium-independent and only blocked by paxilline [15].

**Fig. 3:**
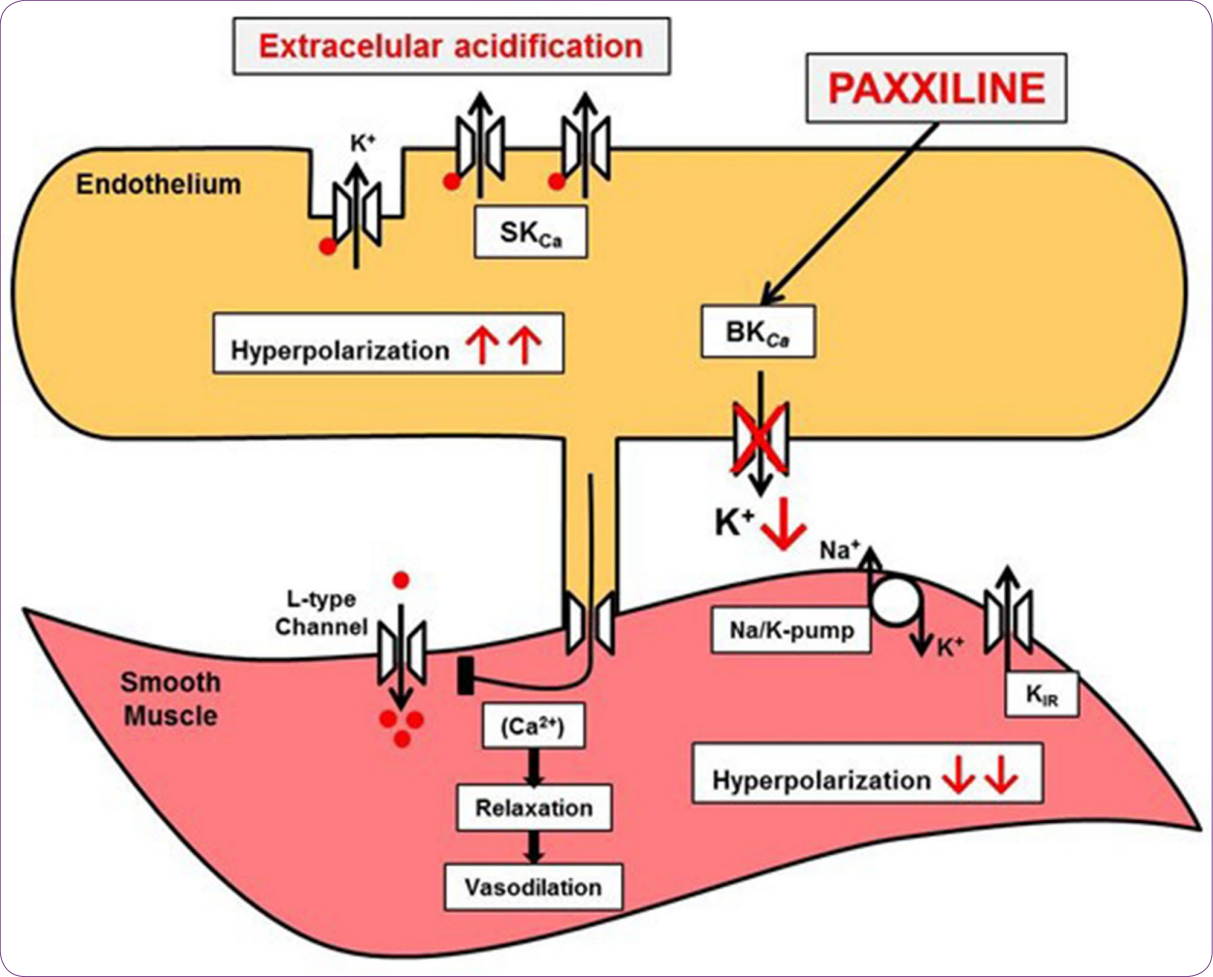
Overall scheme possible inhibition of BK_Ca_ channels by paxilline

In conclusion, high conductance potassium channel (BK_Ca_) activation induced by acid exposure is endothelium-dependent (Fig. 3).

## Abbreviations

PE: phenylephrine
BK_Ca_: calcium-activated potassium channels
KCl: potassium chloride
L-NAME: L-NG-nitroarginine methyl ester
NO: nitric oxide
K_ATP_: ATP-sensitive potassium channel
SK_Ca_: calcium-activated SK potassium channel
cGMP: cyclic guanosine monophosphate
